# HER-2-Positive Ampullary Adenocarcinoma: A Case Report

**DOI:** 10.1089/crpc.2015.29004.koh

**Published:** 2015-11-01

**Authors:** Kevin O'Hayer, John Farber, Charles J. Yeo, Ashwin R. Sama

**Affiliations:** ^1^ Department of Medical Oncology, Thomas Jefferson University Hospital, Philadelphia, Pennsylvania.; ^2^ Department of Pharmacology and Experimental Therapeutics, Thomas Jefferson University Hospital, Philadelphia, Pennsylvania.; ^3^ Department of Pathology, Anatomy, and Cell Biology, Thomas Jefferson University Hospital, Philadelphia, Pennsylvania.; ^4^ Department of Surgery, The Jefferson Pancreas, Biliary, and Related Cancer Center, Thomas Jefferson University, Philadelphia, Pennsylvania.

**Keywords:** ampullary, ctDNA, HER-2

## Abstract

**Background:** Ampullary adenocarcinomas are a rare subset of periampullary tumors with an overall poor prognosis. Treatment decisions are generally extrapolated from pancreatic chemotherapy protocols and consist mainly of traditional chemotherapy drugs. There are no known targets for therapeutic intervention in ampullary adenocarcinoma at this time. Next generation sequencing and other novel molecular profiling of tumors, including circulating tumor DNA (ctDNA), have recently made it possible to better understand tumor biology and elucidate driver mutations which are amenable to targeted therapy. This case describes the use of novel DNA sequencing technology to provide a targeted treatment option, HER-2 inhibition, in a patient with HER-2 overexpressing ampullary adenocarcinoma. This is the first time this has been described in the literature.

**Case presentation:** The patient is a 63-year-old Caucasian man who initially presented with symptoms of obstructive jaundice and was found to have a periampullary tumor. He underwent resection of his tumor and pathology confirmed a stage IIB ampullary adenocarcinoma. He unfortunately developed a recurrence in the liver and lung two years later. Next generation sequencing of his tumor at the time of resection as well as ctDNA analysis demonstrated a HER-2 overexpressing tumor. Following first line therapy with FOLFOX he had progression and was treated with trastuzumab and pertuzumab with stabilization of his disease prior to his ultimate demise from multifocal pneumonia.

**Conclusion:** The use of next generation sequencing as well as ctDNA technology generated a novel therapeutic intervention in our patient. As these techniques become more widespread, it is likely more targeted therapies will be used in these difficult to treat diseases.

## Introduction

Ampullary adenocarcinomas are a minority subset of periampullary carcinomas (PAC), which include tumors of the pancreatic head, distal common bile duct, duodenum, and ampulla of Vater. Although PACs are located in close proximity to one another, each has distinct pathological findings, tumor biology, treatment strategy, and clinical outcomes. Ampulla of Vater tumors are the second most common PAC; however, they account for <1% of all gastrointestinal malignancies. The biology of these tumors more closely resembles that of small intestinal tumors than those of pancreatobiliary origin. Histologically, they appear more adenomatous and patients with familial adenomatous polyposis have a higher incidence of ampullary carcinoma, suggesting a common mechanism with colorectal tumorigenesis. Furthermore, most ampullary carcinomas present while still resectable, have less perineural and lymphovascular invasion, and have improved outcomes compared to tumors of pancreatobiliary origin. Unlike pancreatic tumors, KRAS mutations are less frequent and there is increased COX-2 expression, which is more consistent with intestinal biology. The 5-year postresection survival rate of ampullary tumors (40%) is between duodenal (60%) and pancreatobiliary (20%) tumors. Given the biological differences between ampullary carcinoma and tumors of pancreatobiliary origin, there is debate regarding the appropriate adjuvant treatment of ampullary carcinoma. Generally, treatment is extrapolated from the ESPAC-3 trial, which showed benefit of chemotherapy in high-risk tumors.^1^ Most clinicians prefer to use gemcitabine for adjuvant treatment based on the ESPAC-3 trial; however, FOLFOX is also used in some centers. Currently, there are no known molecular targets with proven therapeutic efficacy in the treatment of ampullary adenocarcinoma.

Novel techniques for tumor sequencing, including circulating tumor DNA (ctDNA) sequencing, have provided new methods to sample patients' tumors and determine whether targetable mutations are present, assess response to therapy, and potentially detect overall tumor burden. In this report, we will describe how the use of this technology led to the discovery of HER-2 amplification in a patient with ampullary adenocarcinoma. Although this amplification is not a common event in ampullary tumors, the finding of HER-2 amplification led to an opportunity to use a targeted therapy with efficacy in other tumor types and ultimately a tumor response for the patient.

## Presentation of Case

The patient is a 63-year-old man who initially presented with obstructive jaundice in the summer of 2010. He underwent an MRI scan which demonstrated a periampullary mass with no evidence of definitive metastatic disease. His CEA and CA 19-9 were both elevated at 4.7 ng/mL and 71 U/mL, respectively. He was transferred to our hospital for further workup and management of his periampullary mass. Endoscopic biopsy of this mass confirmed an intramucosal adenocarcinoma with high-grade dysplasia. He was recommended to undergo pylorus-preserving pancreaticoduodenectomy. His pathology demonstrated a 1.2 × 1.1 × 1 cm invasive moderately differentiated adenocarcinoma arising in association with a tubulovillous adenoma from the ampulla of Vater with invasion into the duodenal wall. The resection margins were negative for tumor. There was no evidence of lymphovascular invasion or perineural invasion. One of the seven specimen lymph nodes was positive for adenocarcinoma. His final staging was pT2pN1M0 or stage IIB ampullary adenocarcinoma. Following surgery, his tumor markers remained elevated with a CA 19-9 of 149 U/mL. The patient's tumor was sent for next-generation sequencing, which revealed a wild-type KRAS and HER-2 amplification by FISH.

**FIG. 1.**
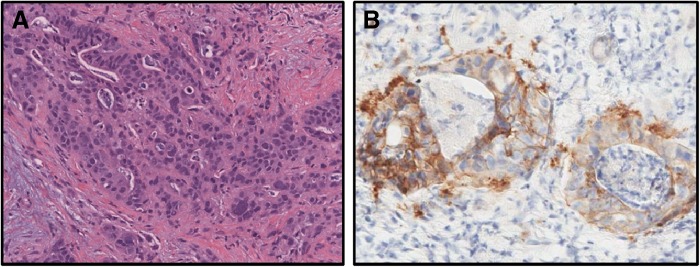
**(A)** Hematoxylin and eosin stain of ampullary carcinoma in liver biopsy. **(B)** HER-2 staining of ampullary carcinoma in liver biopsy.

Following resection of his primary tumor, he received nine cycles of adjuvant 5-FU, leucovorin, and oxaliplatin (FOLFOX). His computed tomography (CT) scans after chemotherapy showed no evidence of recurrent or metastatic disease. He then received chemoradiotherapy with capecitabine radiosensitization and a total of 50.4 Gy external beam radiotherapy to the tumor bed and regional nodes. Following adjuvant therapy, his tumor markers fell into the normal range. He underwent routine follow-up with no evidence of disease until March 2014. At this time, CT scan demonstrated new subcentimeter lesions in the right middle lobe of the lung as well as a subcentimeter right hepatic lobe lesion, which were not amenable to biopsy. PET scan in June 2014 demonstrated metabolically active lesions in both the lung and liver consistent with metastatic disease. Given the inability to biopsy the lesions, the patient's blood was sent for ctDNA analysis. ctDNA results again confirmed the presence of wild-type KRAS as well as HER-2 amplification seen in his previous next-generation sequencing. He subsequently underwent biopsy of an enlarging liver lesion, which was positive for adenocarcinoma consistent with metastatic ampullary carcinoma. HER-2 immunohistochemistry (IHC) demonstrated 80% strong positive staining in this liver biopsy sample (Fig. 1).

The patient received first-line metastatic treatment with FOLFOX beginning in October 2014. He completed nine cycles, which was complicated by neuropathy. CT scans in February 2015 demonstrated increasing size of pulmonary nodules with new hilar and mediastinal lymphadenopathy consistent with progression of disease and FOLFOX was discontinued. Based on the HER-2 amplification seen in next-generation sequencing, ctDNA analysis, and IHC, the decision was made to administer second-line treatment using combined HER-2 inhibition with trastuzumab and pertuzumab. At the time, there were no open trials for HER-2-positive ampullary carcinoma patients and we did not have any open “basket trials.” Insurance approved an off-label use of trastuzumab and pertuzumab based on extrapolated data from the breast cancer CLEOPATRA trial and use of trastuzumab in the gastric cancer ToGA trial.^2,3^ Taxanes were omitted from the regimen because of residual neuropathy from previous oxaliplatin exposure.

**FIG. 2.**
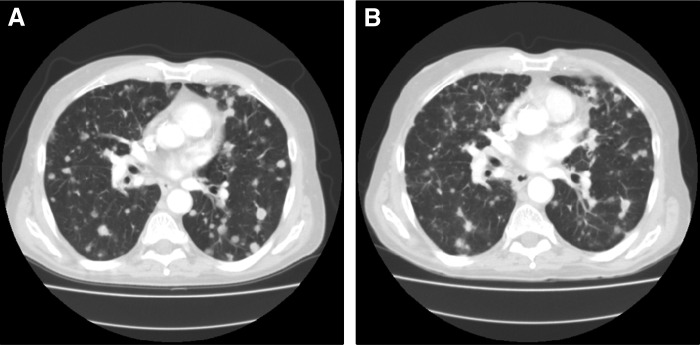
Chest computed tomography scans from January 2005 **(A)** and April 2015 **(B)** demonstrating interval improvement in lesions following initiation of trastuzumab and pertuzumab.

The patient initiated treatment with trastuzumab and pertuzumab every 3 weeks beginning in February 2015. He received four cycles without complication or any new side effects and he continued to work full time. His interim CT scans in April 2015 demonstrated radiological improvement in disease, which did not meet the RECIST criteria for a partial response to therapy and, as such, was termed stable disease (Fig. 2). A 1.3 cm lung lesion decreased to 1.1 cm, a second 1.3 cm lung lesion measured 0.8 cm, and a 2.4 × 2.5 cm liver lesion decreased to 2.2 × 2.2 cm. He went on to complete five cycles of this second-line therapy without incident.

After six cycles of treatment, he was admitted to the hospital with shortness of breath and hypoxia. He was unfortunately found to have progression of pulmonary metastatic disease as well as diffuse ground glass opacities consistent with an infectious or inflammatory process. Although aggressive treatment was pursued, including broad spectrum antibiotic therapy and respiratory support, the patient expired secondary to hypoxic respiratory failure.

## Discussion

This case report details the use of next-generation sequencing along with ctDNA analysis and IHC confirmation of an ampullary carcinoma with HER-2 overexpression. A study of 104 resected ampullary carcinomas demonstrated HER-2 overexpression in 13% of tumors.^4^ This, however, is the first case report of an ampullary carcinoma patient with HER-2 amplification treated with anti-HER-2 therapy. Although the response was short in duration (<4 months) and did not meet the RECIST criteria for a partial response, it is clear from interval imaging that the patient did have stable to improved disease burden with the combination of trastuzumab and pertuzumab with no additional toxicity. This compares favorably with known progression-free survival rates of second-line pancreatic cancer treatments of ∼2 months. It further represents one of the first molecular targets for potential treatment in the relatively small subset of HER-2 amplified ampullary carcinomas.

The effectiveness of trastuzumab and pertuzumab in this patient further underscores the need for future investigations into anti-HER-2 therapy in this population. This regimen was extrapolated from the CLEOPATRA trial in metastatic breast cancer.^3^ Future controlled studies will need to be undertaken to further validate this hypothesis. Basket trials, including MATCH and IMPACT, are testing the hypothesis that molecular markers predict response to a targeted therapy independent of tumor histology and may yield important clinical data moving forward.

Finally, this case report demonstrates the power of ctDNA not only to assess mutational burden in a relatively noninvasive manner but also to discover the amplification of HER-2. In this case, the discovery of HER-2 amplification in the ctDNA sample led to additional biopsy and immunohistochemical confirmation of HER-2 expression. As ctDNA becomes further integrated into the molecular characterization of tumors, it is likely that more patients will receive treatment with targeted agents.

## Abbreviations Used

ctDNA: circulating tumor DNA
CT: computed tomography
IHC: immunohistochemistry
PAC: periampullary carcinomas
